# Effects of Kiwifruit Rootstocks with Opposite Tolerance on Physiological Responses of Grafting Combinations under Waterlogging Stress

**DOI:** 10.3390/plants11162098

**Published:** 2022-08-12

**Authors:** Danfeng Bai, Zhi Li, Shichao Gu, Qiaohong Li, Leiming Sun, Xiujuan Qi, Jinbao Fang, Yunpeng Zhong, Chungen Hu

**Affiliations:** 1 Key Laboratory for Fruit Tree Growth, Development and Quality Control, Zhengzhou Fruit Research Institute, Chinese Academy of Agricultural Sciences, Zhengzhou 450009, China; 2 Key Laboratory of Horticultural Plant Biology, College of Horticulture & Forestry Science, Huazhong Agricultural University, Wuhan 430070, China; 3 Kiwifruit Breeding and Utilization Key Laboratory of Sichuan Province, Sichuan Provincial Academy of Natural Resource Sciences, Chengdu 610015, China

**Keywords:** kiwifruit, scion–rootstock combination, waterlogging tolerance, physiological response, gene expression

## Abstract

Kiwifruit is commonly sensitive to waterlogging stress, and grafting onto a waterlogging-tolerant rootstock is an efficient strategy for enhancing the waterlogging tolerance of kiwifruit plants. KR5 (*Actinidia valvata*) is more tolerant to waterlogging than ‘Hayward’ (*A. deliciosa*) and is a potential resistant rootstock for kiwifruit production. Here, we focused on evaluating the performance of the waterlogging-sensitive kiwifruit scion cultivar ‘Zhongmi 2′ when grafted onto KR5 (referred to as ZM2/KR5) and Hayward (referred to as ZM2/HWD) rootstocks, respectively, under waterlogging stress. The results showed ‘Zhongmi 2′ performed much better when grafted onto KR5 than when grafted onto ‘Hayward’, exhibiting higher photosynthetic efficiency and reduced reactive oxygen species (ROS) damage. Furthermore, the roots of ZM2/KR5 plants showed greater root activity and energy supply, lower ROS damage, and more stable osmotic adjustment ability than the roots of ZM2/HWD plants under waterlogging stress. In addition, we detected the expression of six key genes involved in the kiwifruit waterlogging response mechanism, and these genes were remarkably induced in the ZM2/KR5 roots but not in the ZM2/HWD roots under waterlogging stress. Moreover, principal component analysis (PCA) further demonstrated the differences in the physiological responses of the ZM2/KR5 and ZM2/HWD plants under waterlogging stress. These results demonstrated that the KR5 rootstock can improve the waterlogging tolerance of grafted kiwi plants by regulating physiological and biochemical metabolism and molecular responses.

## 1. Introduction

Adverse environmental factors including salinity [1], alkalinity [2], drought [3], and high and low temperatures [4,5] can seriously inhibit the growth and development of some horticultural crops and are associated with substantial economic losses. Waterlogging stress, caused by continuous or excessive rain and poor soil drainage, is a constraint for plant growth and development [6]. Statistically, >1700 Mha of land worldwide suffers from waterlogging every year [7]. Hypoxia and even anoxia around roots, caused by waterlogging stress, is the main constraint for plant survival and growth [8], impacting plant physiology and biochemical metabolism, and ultimately causing plant death [8,9,10]. To cope with waterlogging stress, plants have developed several mechanisms including the formation of aerial roots [11], changes in respiration patterns [12], and the scavenging of reactive oxygen species (ROS) [13].

Plant tolerance to abiotic stress is a complex trait involving several environmental factors, and improving the tolerance of agricultural crops using modern breeding and biotechnological approaches has proven to be difficult [14]. Grafting, an ancient and traditional method of reproducing plants by connecting a scion and rootstock [15,16], can modify the traits of the aerial parts of a plant, including resistance to stress, thereby increasing yield and improving fruit quality [17,18,19]. Many studies have confirmed that the resistance of commercial plants to abiotic stress can be improved by grafting resistant rootstocks [17,20,21]. For example, in the rootstock of Carrizo citrange (*Citrus sinensis* × *Poncirus trifoliata*), the resistance of grafted plants to drought and heat stress combinations could be improved by modifying the scion antioxidant system [22]. Moreover, it has been shown that grafting can improve the waterlogging tolerance of grafted plants. For instance, a study on apple tree waterlogging tolerance demonstrated that trees grafted onto waterlogging-tolerant rootstock CG4814 were more resistant to waterlogging stress than those grafted onto other sensitive rootstocks [23]. Calogero Iacona et al. [24] also demonstrated that the flooding tolerance of peach cultivars can be improved by using the S.4 clone rootstock.

Kiwifruit (*Actinidia* spp.) is popular among consumers because of its rich vitamin content and unique taste [25]. However, kiwifruit plants are extremely sensitive to waterlogging stress because of their high transpiration rate and fleshy roots [26,27], which largely increases its planting risk in waterlogged soils. Kiwifruit is generally propagated via grafting. Currently, New Zealand, Italy, and other kiwifruit-producing countries generally select seedlings of kiwifruit varieties from *A. deliciosa* as rootstocks, such as ‘Bruno’ and Hayward [28,29,30]. In China, seedlings of ‘Miliang’ (*A. deliciosa*), ‘Qinmei’ (*A. deliciosa*), and some wild kiwifruit plants are widely used as rootstocks for kiwifruit production [31,32,33]. However, the rootstocks from *A. chinensis* and *A. deliciosa* are commonly considered to be sensitive to waterlogging stress [26,34]. Therefore, there is an urgent need to screen waterlogging-tolerant rootstocks and evaluate their effects on the waterlogging tolerance of grafted kiwifruit plants. To date, most studies on the waterlogging tolerance mechanism of kiwifruit have focused on the screening and evaluation of resistant rootstocks. However, the tolerance mechanism of grafted kiwifruit plants to waterlogging stress has not been explored in detail.

Previously, our research demonstrated that the kiwifruit rootstock KR5 (*A. valvata*) is more tolerant than ‘Hayward’ (*A. deliciosa*) to waterlogging stress [35], and identified some key genes involved in the waterlogging tolerance mechanism based on transcriptome data, including *ADH1* (i1_LQ_K_c67155/f1p0/1459), *ADH2* (i1_LQ_K_c38965/f1p0/1342), *MnSOD1743* (i1_LQ_K_c14090/f1p1/1743), *POD1591* (i1_HQ_K_c28263/f2p2/1591), *ERF73* (i1_HQ_K_c88560/f4p0/1107), and *ERF78* (i1_HQ_K_c68003/f14p0/1110) [36]. In the present study, we grafted ‘Zhongmi 2′ (*A. deliciosa*), a waterlogging-sensitive scion cultivar from the Zhengzhou Fruit Research Institute (ZFRI), Chinese Academy of Agricultural Sciences (CAAS) [37], onto KR5 and ‘Hayward’ to obtain two scion–rootstock combinations, referred to as ZM2/KR5 and ZM2/HWD, respectively. To investigate the effect of using rootstocks with opposite resistance to waterlogging tolerance on kiwifruit growth, we performed waterlogging experiments and determined the physiological and biochemical changes, and associated gene expression, for the different scion–rootstock combinations. We specifically sought to verify the function of the KR5 rootstock in the improvement in the waterlogging tolerance of the investigated scion–rootstock combinations, providing a theoretical basis for the breeding, popularization, and application of waterlogging-tolerant rootstocks in kiwifruit cultivation.

## 2. Results

### 2.1. Phenotype, Survival Rate, and Root Activity of Two Rootstock–Scion Combinations under Waterlogging Stress

**Figure 1:**
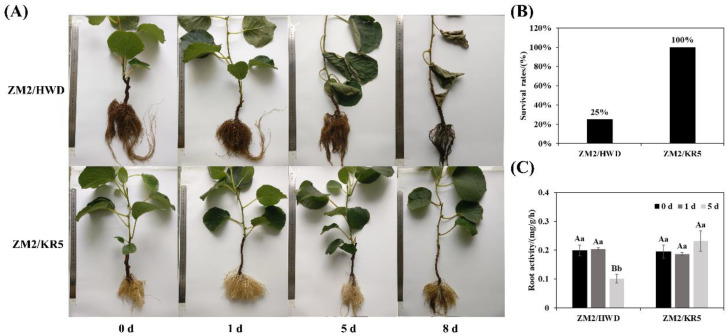
(**A**) Phenotype, (**B**) survival rate, and (**C**) root activity of two kiwifruit scion–rootstock combinations under waterlogging stress. ZM2/HWD and ZM2/KR5 indicate grafting ‘Zhongmi 2′ onto ‘Hayward’ and KR5 rootstocks, respectively. Data are the mean values ± SD (*n* = 3). Capital letters denote significant differences between the different waterlogging treatment stages for each scion–rootstock combination, and lower-case letters denote significant differences among the scion–rootstock combinations within the different waterlogging treatment stages according to a least significant difference (LSD) test (*p* ≤ 0.05).

Waterlogging damaged the growth of ‘Zhongmi 2′ scions grafted on ‘Hayward’ and KR5 rootstocks (Figure 1a). After being waterlogged for 1 d, ZM2/HWD and ZM2/KR5 plants both grew normally. However, after 5 d, different morphological responses were observed. Notably, the feeder roots of waterlogged ZM2/HWD plants decayed, leaves wilted and necrotized, and the plants defoliated, whereas most of the fibrous roots survived. In contrast, the aerial and underground parts of the ZM2/KR5 plants performed well after waterlogging for 5 d. The lower roots of the ZM2/KR5 plants turned black but did not rot until day 8, while most of the ZM2/HWD plants had died by this time, with a survival rate of only 25% (Figure 1b). Furthermore, the root activity of the ZM2/HWD plants decreased significantly after 5 d of waterlogging treatment (*p* < 0.05), while the ZM2/KR5 plants presented stable root activity (Figure 1c).

### 2.2. Effects of Waterlogging Stress on the Photosynthesis of Scion–Rootstock Combinations

**Figure 2:**
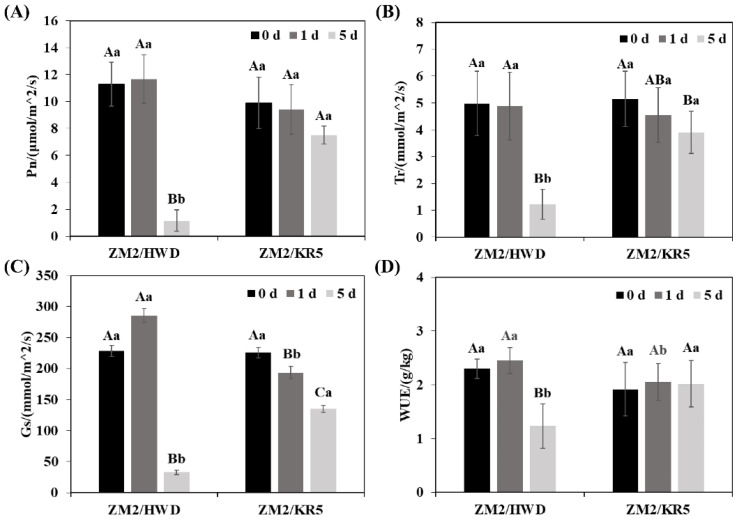
(**A**) Net photosynthesis (Pn), (**B**) transpiration rate (Tr), (**C**) stomatal conductance (Gs), and (**D**) water use efficiency (WUE) of ZM2/HWD and ZM2/KR5 kiwifruit plants under waterlogging stress. ZM2/HWD and ZM2/KR5 indicate grafting ‘Zhongmi 2′ onto ‘Hayward’ and KR5 rootstocks, respectively. Data are the mean values ± SD (*n* = 3). Capital letters denote significant differences between the different waterlogging treatment stages for each scion–rootstock combination, and lower-case letters denote significant differences among the scion–rootstock combinations within the different waterlogging treatment stages according to a least significant difference (LSD) test (*p* ≤ 0.05).

As the ZM2/HWD plants died when exposed to being waterlogged for 8 d, the leaf net photosynthetic efficiency (Pn), transpiration rate (Tr), stomatal conductance (Gs), and water use efficiency (WUE) of the grafted plants were measured on days 0, 1, and 5 (Figure 2). The Pn, Tr, Gs, and WUE of the ZM2/HWD plant leaves continuously decreased during waterlogging stress, and after 5 d, had decreased by 89.7%, 75.6%, 85.6%, and 46.4%, respectively, compared with those of the control (*p* < 0.05). Although the Pn, Tr, Gs, and WUE of the ZM2/KR5 plant leaves were also inhibited under waterlogging stress, the reductions in these metrics were more pronounced and not significant in comparison to those of the control plants.

### 2.3. Effects of Waterlogging Stress on the O_2_^−^ Production Rate, H_2_O_2_ Content, and MDA Content in Different Scion–Rootstock Combinations

**Figure 3:**
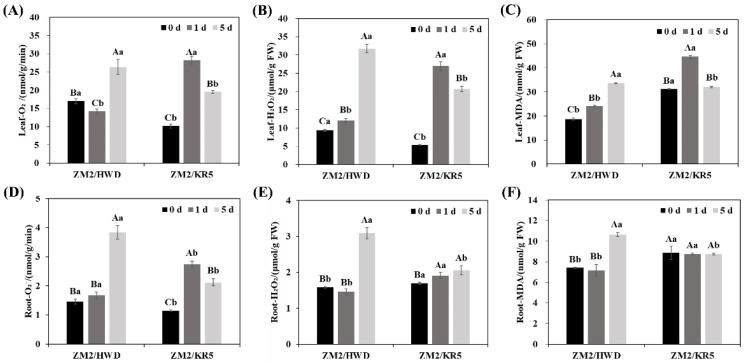
(**A**,**D**) Superoxide anion (O_2_^−^) production rate in leaves and roots, (**B**,**E**) hydrogen peroxide (H_2_O_2_) content in leaves and roots, and (**C**,**F**) malondialdehyde (MDA) content in leaves and roots. ZM2/HWD and ZM2/KR5 indicate grafting ‘Zhongmi 2′ onto ‘Hayward’ and KR5 rootstocks, respectively. Data are the mean values ± SD (*n* = 3). Capital letters denote significant differences between the different waterlogging treatment stages for each scion–rootstock combination, and lower-case letters denote significant differences among the scion–rootstock combinations within the different waterlogging treatment stages according to a least significant difference (LSD) test (*p* ≤ 0.05).

In the ZM2/HWD plants, the superoxide anion (O_2_^−^), hydrogen peroxide (H_2_O_2_), and malondialdehyde (MDA) contents continued to increase during the waterlogging treatment (Figure 3); after 5 d, increases of 55.1%, 238.1%, and 79.9% and 162.5%, 94.9%, and 43.3% were observed in leaves and roots, respectively, compared with those in the control plants. In the ZM2/KR5 plants, the levels of O_2_^−^, H_2_O_2_, and MDA in the leaves and O_2_^−^ in the roots first increased sharply and then decreased during waterlogging stress (Figure 3a–d). After 5 d of waterlogging treatment, the H_2_O_2_ content in the ZM2/KR5 roots increased by 21.2% compared with that in the roots of the control plants (Figure 3e). Moreover, the MDA content of ZM2/KR5 roots remained stable throughout the waterlogging stress treatment, with no difference compared with that of the control plants after 5 d (Figure 3f). These results indicated that using the KR5 rootstock can protect the leaves of grafted plants against oxidative stress induced by waterlogging stress.

### 2.4. Physiological Response of Grafted Plant Roots to Waterlogging Stress

**Figure 4:**
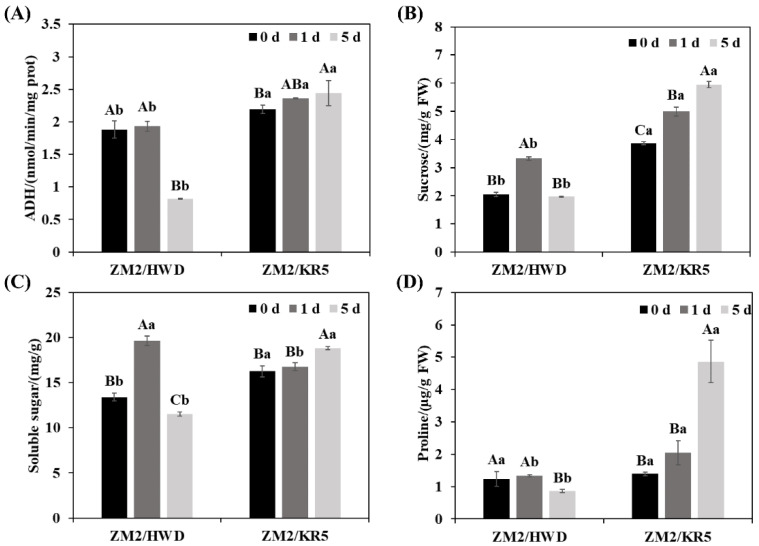
Changes in (**A**) ADH enzyme activity, (**B**) sucrose, (**C**) soluble sugar, and (**D**) proline in the roots of different scion–rootstock combinations under waterlogging stress. ZM2/HWD and ZM2/KR5 indicate grafting ‘Zhongmi 2′ onto ‘Hayward’ and KR5 rootstocks, respectively. Data are the mean values ± SD (*n* = 3). Capital letters denote significant differences between the different waterlogging treatment stages for each scion–rootstock combination, and lower-case letters denote significant differences among the scion–rootstock combinations within the different waterlogging treatment stages according to a least significant difference (LSD) test (*p* ≤ 0.05).

To study the response of the roots of the grafted plants to waterlogging stress, we detected physiological indicators, including alcohol dehydrogenase (ADH) activity, sucrose content, soluble sugar content, and proline content (Figure 4). ADH is a key enzyme involved in the anaerobic fermentation pathway, and ADH enzymes can generate energy under anaerobic conditions. Notably, the trends in ADH activity in the ZM2/HWD and ZM2/KR5 plants were inconsistent (Figure 4a). For example, after 5 days of waterlogging treatment, ADH activity in the ZM2/HWD plants decreased by 56.6%, while that in the ZM2/KR5 plants increased by 11.6%. The observed changes in sucrose, soluble sugar, and proline under waterlogging stress were similar for the scion–rootstock combinations (Figure 4b–d). For the ZM2/HWD plants, the sucrose, soluble sugar, and proline contents first increased and then decreased, with a total decrease of 40.6%, 41.3%, and 35.3%, respectively, after 5 d relative to that on day 1 of the experiment. In contrast, the corresponding changes in the ZM2/KR5 plants were increases of 53.7%, 15.9%, and 248.4%, respectively.

### 2.5. Molecular Response of Grafted Plant Roots to Waterlogging Stress

**Figure 5:**
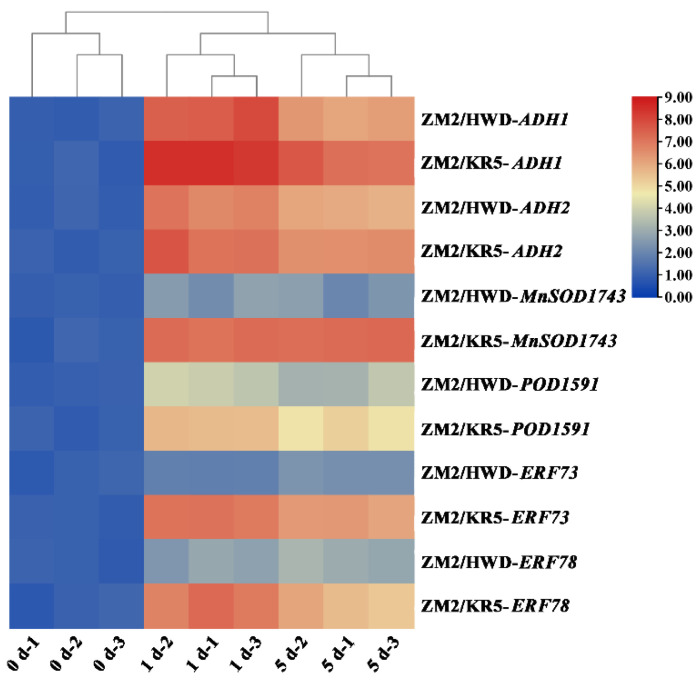
Expression profiles of waterlogging stress-related genes in the roots of scion–rootstock combinations. Blue color indicates a low expression level, whereas red color indicates a high expression level. Heat map was created using Tbtools.

To further explore root molecular responses under waterlogging stress, we performed quantitative real-time PCR (qRT-PCR) to analyze the expression of key genes in the roots of the two scion–rootstock combinations (Figure 5). We found that *ADH1* and *ADH2* were significantly induced in the roots of the two grafted plants under waterlogging stress; however, the increase was greater in the ZM2/KR5 plants. Two antioxidant enzyme genes, *MnSOD1743* and *POD1591*, were also induced significantly in the ZM2/KR5 roots, but no significant difference was observed in the ZM2/HWD roots. Similarly, *ERF73* and *ERF78*, two ERF-VII members from *A. valvata*, were more strongly expressed under waterlogging stress in the ZM2/KR5 plants than in the sensitive ZM2/HWD plants.

### 2.6. Principal Component Analysis (PCA)

**Figure 6:**
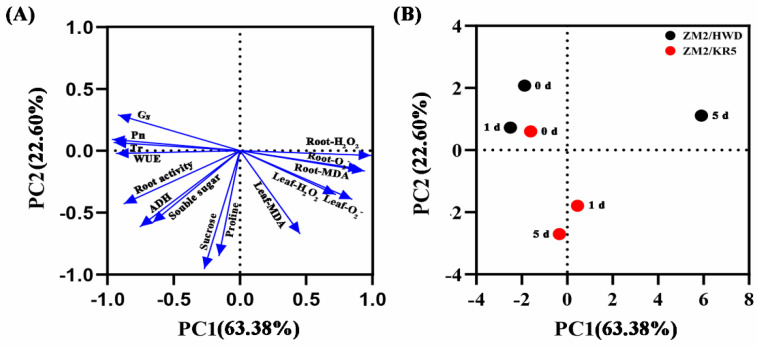
(**A**) PCA loading plot and (**B**) score plot showing the effects of two different rootstocks on the waterlogging tolerance of scion–rootstock combinations. ZM2/HWD and ZM2/KR5 indicate grafting ‘Zhongmi 2′ onto ‘Hayward’ and KR5 rootstocks, respectively.

PCA was performed to evaluate the overall effects of the two rootstocks with different waterlogging tolerances on the waterlogging tolerance of the scion–rootstock combinations (Figure 6). The first two principal components (PCs) explained approximately 85.98% of the total variance. PC1, which explained 63.38% of the variance, had positive associations with Leaf-O_2_^−^, Leaf-H_2_O_2_, Leaf-MDA, Root-O_2_^−^, Root-H_2_O_2_ and Root-MDA content. Variances in the photosynthetic indices (Pn, Tr, Gs, and WUE) were positively associated with PC2, which explained 22.60% of the total variance. Root activity, sucrose, soluble sugar, proline, and anaerobic fermentation enzyme ADH activity were negatively associated with PC1 and PC2. The short distance between the control groups showed that there was little difference between the two types of grafted plants before waterlogging, whereas after waterlogging treatment, the two types of plants could be separated based on PC1 or PC2. This indicates that rootstocks with different tolerances affected the response of grafted plants when exposed to waterlogging stress.

## 3. Discussion

The expansion of kiwifruit cultivation is restricted in waterlogged areas because of the low tolerance of this plant to waterlogging stress. When waterlogged, the root tips of plants were damaged initially because of oxygen deficiency, thereby affecting nutrient and water uptake, leading to plant wilting and even death in severe cases [8,38]. In the present study, the roots of ZM2/HWD plants decayed after 5 d of waterlogging, associated with leaf wilting, necrosis, and defoliation. After 8 d of waterlogging, the mortality rate of the ZM2/HWD plants was as high as 75%. In contrast, the roots of the ZM2/KR5 plants grew well during waterlogging stress. Importantly, root activity can indicate the ability of roots to absorb nutrients and water [39]. Thus, under waterlogging stress, the root activity of the ZM2/HWD plants significantly decreased, while that of the ZM2/KR5 plants was stable at a much higher level.

The main pathway of gas exchange between plant leaves and the external environment is the stomata, and this process reflects the metabolic activities of plants [40], with stomatal closure often being the first response to waterlogging stress [41]. In our study, the stomatal conductance (Gs) of the two scion–rootstock combinations significantly decreased after 5 d of waterlogging. This led to a decline in the photosynthetic indices including the leaf net photosynthetic efficiency (Pn), transpiration rate (Tr), and water use efficiency (WUE). This finding indicates that the metabolic activity of the ZM2/HWD and ZM2/KR5 leaves was constrained on their exposure to the waterlogging stress, which is similar to the results of previous studies [42]. Most notably, the inhibitory effect of waterlogging on photosynthesis was much weaker in the ZM2/KR5 plants than that in the ZM2/HWD plants.

The negative effects of waterlogging on roots mainly occur via (1) an energy crisis that causes anaerobic respiration, and (2) an imbalance between ROS accumulation and scavenging [43]. At low oxygen concentrations, aerobic respiration is inhibited, and anaerobic respiration is undertaken by anaerobic respiration enzymes [12]. ADH is a key enzyme in the anaerobic respiration pathway that converts acetaldehyde to ethanol, thereby providing energy to plants and preventing acetaldehyde toxicity [44]. In the present study, ZM2/KR5 roots had higher ADH activity and sucrose content relative to the ZM2/HWD roots, indicating that these plants could maintain metabolism and avoid energy crisis when exposed to waterlogging stress. In addition, the ZM2/KR5 roots experienced less ROS damage than the ZM2/HWD roots. Interestingly, the leaves of the ZM2/KR5 plants also suffered less ROS damage than the leaves of ZM2/HWD plants. These results further demonstrated that ROS scavenging was improved by using a tolerant rootstock, which is consistent with previous research on citrus plants [22].

When subjected to waterlogging stress, tolerant plants can change their intracellular water potential by rapidly accumulating osmotic regulatory substances, such as soluble sugar and proline, over a short period to deal with stress [45]. We found that the content of soluble sugar and proline in the ZM2/HWD roots was significantly lower than that in the control plants after 5 d of waterlogging treatment. In contrast, the soluble sugar and proline contents of the ZM2/KR5 roots increased continuously during waterlogging stress. A higher soluble sugar and proline content can ensure plant tolerance against the osmotic stress caused by waterlogging.

An increase in the transcript abundance of the *ADH* genes that control ADH enzyme synthesis under waterlogging stress has been confirmed in many studies [46,47]. Here, we detected the expression levels of *ADH1* (i1_LQ_K_c67155/f1p0/1459) and *ADH2* (i1_LQ_K_c38965/f1p0/1342) involved in anaerobic respiration under waterlogged conditions. We found that the expression of *ADH1* and *ADH2* in the ZM2/KR5 roots was higher than that in the ZM2/HWD roots. This was further verified by the higher ADH enzyme activity of the ZM2/KR5 roots. In addition, to maintain the balance between ROS accumulation and scavenging in adverse environments, plants have developed an antioxidant defense system comprising of SOD, POD, and CAT enzymes [48,49]. Based on the qRT-PCR assay, we found that the expression of two key antioxidant enzymes, *MnSOD1743* (i1_LQ_K_c14090/f1p1/1743) and *POD1591* (i1_HQ_K_c28263/f2p2/1591), was higher in the ZM2/KR5 roots than that in the ZM2/HWD roots. This finding further demonstrated the ROS scavenging ability of the ZM2/KR5 roots under waterlogging stress.

The ERF (Ethylene Responsive Factor) family plays an important role in the abiotic stress response, including drought [50], low temperature [51], and salt stress [52,53]. Moreover, ERF-VII members have been demonstrated to be crucial for plant defense mechanisms in response to waterlogging stress [54]. In our study, two ERF-VII members, *ERF73* (i1_HQ_K_c88560/f4p0/1107) and *ERF78* (i1_HQ_K_c68003/f14p0/1110), were remarkably induced in the ZM2/KR5 roots under waterlogging treatment, but they were almost absent in the roots of the ZM2/HWD plants. This is consistent with previous conclusions that indicated that *ERF73* and *ERF78* play crucial roles in the molecular-scale defense mechanism of plants under waterlogging stress [55].

## 4. Materials and Methods

### 4.1. Plant Materials, Waterlogging Treatment, and Sampling

This study was carried out at the ZFRI, CAAS (latitude 34°43′ N, longitude 113°39′ E, and altitude 111 m). To ensure the consistency of plant materials, two-year-old tissue culture plants of ‘Hayward’ (*A. deliciosa*, waterlogging sensitive) and KR5 (*A. valvata*, waterlogging tolerant) were used as rootstocks, and the height of rootstock selected in this study was 15–20 cm, and the stem diameter was 0.6–0.8 cm. Then, *A. deliciosa* cultivar ‘Zhongmi’ 2 was grafted onto the two rootstocks, producing two scion–rootstock combinations Zhongmi 2/Hayward (ZM2/HWD) and Zhongmi 2/KR5 (ZM2/KR5). In total, thirty ZM2/HWD and thirty ZM2/KR5 potted plants were obtained through grafting.

Waterlogging was conducted according to a previously reported method [56]. Two-year-old potted ZM2/HWD and ZM2/KR5 plants were placed in plastic containers (45 cm × 35 cm × 16 cm) filled with water, and the water level was maintained at 2 cm above the soil level. Moreover, prior to the treatments, the terminal shoots and sprouts of vines were pruned to keep their height at approximately 50 cm, only one main shoot with diameter of 0.4–0.6 cm was retained per plant. The grafted plants were subjected to waterlogging stress for 0, 1, 5, and 8 d. The leaves and roots of the waterlogged plants (in triplicate, with one repeat including three plants) were collected, immediately placed in sealed bags, frozen in liquid nitrogen, and stored at −80 °C until analysis.

### 4.2. Determination of Survival Rate and Root Activity

The survival rate of the two scion–rootstock combinations was determined manually after 8 d of waterlogging. Root activity was measured using the triphenyl tetrazolium chloride (TTC) method with some modifications, and expressed in mg/g/h [39]. Briefly, 0.5 g of roots was added to a mixture of 5 mL of TTC (0.4%) and 5 mL of Tris-HCL (pH 7.0). The samples were then subjected to dark treatment at 37 °C for 1 h, after which 2 mL of 1 mol·L^−1^ H_2_SO_4_ was added to terminate the reaction. Finally, the treated root was added to a mortar with ethyl acetate, ground over ice, filtered and diluted to 10 mL. The absorbance value at 485 nm was measured using a spectrophotometer (Thermo fisher scientific, Waltham, MA, USA).

### 4.3. Determination of Photosynthetic Indices

The net photosynthetic efficiency (Pn), transpiration rate (Tr), stomatal conductance (Gs), and water use efficiency (WUE) of six selected plants from each scion–rootstock combination were measured using a portable photosynthesis instrument CI-340 (CID Bio-Science, Washington, WA, USA) between 9:30 and 11:30 a.m. on days 0, 1, 5, and 8 of the waterlogging stress treatment. We marked the six selected grafted plants and performed continuous measurements during the experiment

### 4.4. Determination of O_2_^−^, H_2_O_2_, and MDA Content

The method described by Huang et al. [57] was used to determine the rate of O_2_^−^ generation. Changes in the rate of O_2_^−^ generation (nmol/g/min) were recorded using a spectrophotometer at 530 nm. H_2_O_2_ and MDA levels were determined according to the protocol of Hussain et al. [58]. H_2_O_2_ content was recorded using a spectrophotometer at 415 nm, and the results were expressed as µmol/g of fresh sample weight (FW). MDA content was measured using reactive substances of thiobarbituric acid (RSTBA), and recorded by subtracting at 600 nm from the absorbance value at 532 nm. The results were expressed as nmol/g of fresh sample weight.

### 4.5. Determination of ADH Activity, Sucrose, Soluble Sugar, and Proline Content

ADH (1 U) was defined as the amount of enzyme required to decompose 1 nmol of NADH per minute per mg protein [59]. The sucrose content (mg/g FW) of roots was determined using plant sucrose and soluble protein kits (KeMing, Suzhou, China). ADH enzyme activity was measured at 340 nm using a microplate reader. Soluble sugar content was measured according to a previously reported method using a microplate reader at 625 nm (mg/g FW) [60]. Profile content was determined according to Yao [3] and expressed as µg/g FW.

### 4.6. Total RNA Extraction and qRT-PCR Analyses

Total RNA extraction from the grafted plant roots (control and treatment plants) was performed using a quick RNA isolation kit (HuayueYang Biotechnology, Beijing, China). RNA samples were stored at −80 °C until subsequent analyses. First strand cDNA was synthesized using a First Strand cDNA Synthesis Kit (Novoprotein, Suzhou, China). Previous reported primers were used for qRT-PCR. qRT-PCR experiments were performed in a final volume of 20 µL with the NovoStart^®^SYBR qPCR SuperMix Plus kit (Novoprotein, Suzhou, China) using a LightCycler 480 II (Roche, Basel, Switzerland) on a 96-well plate. Each sample consisted of three technical replicates. The reaction mixture (20 μL) contained 10 μL of super mix, 0.8 μL of cDNA, 0.4 μL of each forward and reverse primers, and 8.4 μL of RNase-free water.

### 4.7. Statistical Analysis

All experiments were performed in triplicate. Analysis of variance, mean comparisons, and data visualization were performed using OriginPro 2022 v 9.9.0.220 (OriginLab Corporation, Northampton, MA, USA). Statistically significant differences were calculated using the LSD test (*p* ≤ 0.05). A heatmap was drawn using TBtools software v1.09861 (Chengjie Chen, Guangzhou, China), a toolkit developed for interactive analyses of big biological datasets [61]. The means of the tested indicators were analyzed using PCA in GraphPad Prism 9.3.1 (GraphPad Software, San Diego, CA, USA).

## 5. Conclusions

This study demonstrated that scion cultivar ‘Zhongmi 2′ (*A. deliciosa*) grafted onto waterlogging-tolerant rootstock KR5 (*A. valvata*) performs better than the same cultivar grafted onto waterlogging-sensitive rootstock ‘Hayward’ (*A. deliciosa*) when exposed to waterlogging stress. Thus, as a rootstock, KR5 can maintain root activity, ensure energy supply, scavenge excessive ROS, and accumulate osmotic substances under waterlogging stress, thereby enhancing the tolerance of the scion to waterlogging stress. In addition, some key genes involved in the waterlogging tolerance mechanism increased remarkably in the ZM2/KR5 plants. PCA analysis further demonstrated the differences in the physiological responses of ZM2/HWD and ZM2/KR5 plants on their exposure to waterlogging stress. Based on our results, the use of more waterlogging-tolerant rootstocks could increase the environmental adaptation of kiwifruit scions, thereby improving performance under waterlogging stress. As such, the selection of stress-resistant rootstocks is important for kiwifruit production under adverse environmental conditions.

## Data Availability

All data generated or analyzed during this study are included in this published article.
